# Ellipticine cytotoxicity to cancer cell lines — a comparative study

**DOI:** 10.2478/v10102-011-0017-7

**Published:** 2011-06

**Authors:** Marie Stiborová, Jitka Poljaková, Eva Martínková, Lucie Bořek-Dohalská, Tomáš Eckschlager, Rene Kizek, Eva Frei

**Affiliations:** 1 Department of Biochemistry, Faculty of Science, Charles University, Prague, Czech Republic; 2 Department of Pediatric Hematology and Oncology, 2nd Medical School, Charles University and University Hospital Motol, Prague, Czech Republic; 3 Department of Chemistry and Biochemistry, Faculty of Agronomy, Mendel University of Agriculture and Forestry, Brno, Czech Republic; 4 Division of Preventive Oncology, National Center for Tumor Diseases, German Cancer Research Center, Heidelberg, Germany

**Keywords:** Ellipticine, cancer cell lines, DNA adducts, mechanims of acticancer effects of ellipticine

## Abstract

Ellipticine is a potent antineoplastic agent exhibiting multiple mechanisms of action. This anticancer agent should be considered a pro-drug, whose pharmacological efficiency and/or genotoxic side effects are dependent on its cytochrome P450 (CYP)- and/or peroxidase-mediated activation to species forming covalent DNA adducts. Ellipticine can also act as an inhibitor or inducer of biotransformation enzymes, thereby modulating its own metabolism leading to its genotoxic and pharmacological effects. Here, a comparison of the toxicity of ellipticine to human breast adenocarcinoma MCF-7 cells, leukemia HL-60 and CCRF-CEM cells, neuroblastoma IMR-32, UKF-NB-3 and UKF-NB-4 cells and U87MG glioblastoma cells and mechanisms of its action to these cells were evaluated. Treatment of all cells tested with ellipticine resulted in inhibition of cell growth and proliferation. This effect was associated with formation of two covalent ellipticine-derived DNA adducts, identical to those formed by 13-hydroxy- and 12-hydroxyellipticine, the ellipticine metabolites generated by CYP and peroxidase enzymes, in MCF-7, HL-60, CCRF-CEM, UKF-NB-3, UKF-NB-4 and U87MG cells, but not in neuroblastoma UKF-NB-3 cells. Therefore, DNA adduct formation in most cancer cell lines tested in this comparative study might be the predominant cause of their sensitivity to ellipticine treatment, whereas other mechanisms of ellipticine action also contribute to its cytotoxicity to neuroblastoma UKF-NB-3 cells.

## Introduction

**Figure 1:**
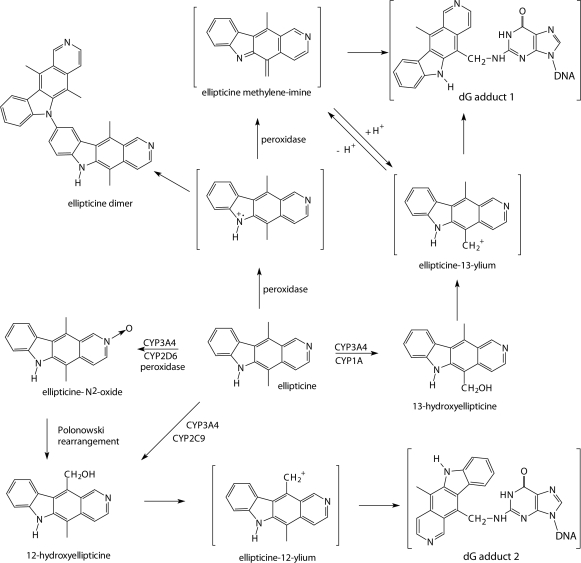
Scheme of the metabolism of ellipticine by peroxidases and human CYPs showing the characterized metabolites and those proposed to form DNA adducts. The compounds shown in brackets are hypothetical electrophilic metabolites postulated as ultimate arylating species or postulated *N* ^2^-deoxyguanosine adducts.

Ellipticine (5,11-dimethyl-6*H*-pyrido[4,3-*b*]carbazole, Figure 1), an alkaloid isolated from *Apocyanaceae* plants, exhibits significant antitumor and anti-HIV activities (for a summary see Stiborová *et al*., 2001). The main reason for the interest in ellipticine and its derivatives for clinical purposes is their high efficiency against several types of cancer, their rather limited toxic side effects, and their complete lack of hematological toxicity (Auclair, 1987). Nevertheless, ellipticine is a potent mutagen. Most ellipticine derivatives are mutagenic to *Salmonella typhimurium* Ames tester strains, bacteriophage T4, *Neurospora crassa*, and mammalian cells and induce prophage lambda in *Escherichia coli* (for an overview see Stiborová *et al.*, 2001).

Ellipticine has been reported to arrest cell cycle progression by regulating the expression of cyclin B1 and Cdc2 as well as phosphorylation of Cdc2 (Kuo *et al*., 2005a; b) to induce apoptotic cell death by generation of cytotoxic free radicals, activation of Fas/Fas ligand system, regulation of Bcl-2 family proteins (Kuo *et al*., 2005a; b; 2006), increase of wild-type p53, rescue of mutant p53 activity and initiation of the mitochondrial apoptosis pathway (Garbett and Graves., 2004; Kuo *et al*., 2005a; b; 2006). Ellipticine also activates the p53 pathway in glioblastoma cells; its impact on these cancer cells depends on the p53 status. In a U87MG glioblastoma cell line expressing p53wt, ellipticine provoked an early G0/G1 cell cycle arrest, whereas in a U373 cell line expressing p53mt it caused arrest in S and G2/M phase (Martínková *et al*., 2010).

Ellipticine and 9-hydroxyellipticine also cause selective inhibition of p53 protein phosphorylation in several human cancer cell lines (Ohashi *et al*., 1995; Sugikawa *et al*., 1999) and this correlates with their cytotoxic activity. However, the precise molecular mechanism responsible for these effects has not been explained yet. Chemotherapy-induced cell cycle arrest was shown to result from DNA damage caused by a variety of chemotherapeutics. In the case of ellipticine, it has been suggested that the prevalent DNA-mediated mechanisms of its antitumor, mutagenic and cytotoxic activities are (i) intercalation into DNA and (ii) inhibition of DNA topoisomerase II activity (Auclair, 1987; Garbett & Graves, 2004; Stiborová *et al*., 2006; 2011).

**Figure 2:**
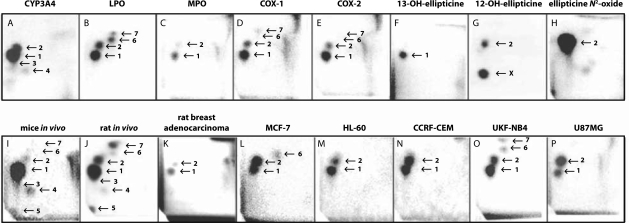
Autoradiographic profiles of ellipticine-derived DNA adducts analyzed with the ^32^P-postlabeling assay. Adduct profiles obtained from calf thymus DNA reacted with ellipticine (100 µM) and CYP3A4 (A), bovine LPO (B), human MPO (C), ovine COX-1 (D), human COX-2 (E), from calf thymus DNA reacted with 13-hydroxyellipticine (F), 12-hydroxyellipticine (G), ellipticine *N* ^2^-oxide (H), from liver DNA of C57BL/6 mice treated i.p. with 10 mg ellipticine per kilogram body weight (I), from liver DNA of Wistar rats treated i.p. with 40 mg ellipticine per kilogram body weight (J), from DNA of breast adenocarcinoma of Wistar rats treated i.p. with 4 mg ellipticine per kilogram body weight (K), from DNA of breast adenocarcinoma MCF-7 cells (L), leukemia HL-60 (M) and CCRF-CEM cells (N), neuroblastoma UK-NB-4 cells (O) and glioblastoma U87MG cells (P) treated with ellipticine. Cancer cells lines were treated with 10 µM ellipticine except of HL-60 cells that were treated with 5 µM ellipticine. Adduct spots 1–7 correspond to the ellipticine-derived DNA adducts. Besides adduct 2 formed by 12-hydroxyellipticine, another strong adduct (spot X in panel G) was generated, which was not found in any other activation systems or *in vivo*. Experimental conditions for panels A–J are described in our previous studies (Stiborová *et al.*, 2004; 2007a) and those for panels L–P in Materials and Methods. Experimental conditions for panel K were as follows: female Wistar rats bearing the *N*-methyl-*N*-nitrosourea induced mammary adenocarcinoma (McCormick *et al*., 1981) were i.p. treated with 4 mg ellipticine per kilogram body weight. Ellipticine was administered dissolved in 1% acetic acid at a concentration of 2.5 mg/ml. One day after ellipticine treatment, DNA from tumor and normal breast tissues (McCormick *et al*., 1981) was isolated and analyzed for formation of DNA adducts using the nuclease P1 version of the ^32^P-postlabeling assay as described (Stiborová *et al*., 2001). All experiments with animal models were conducted in accordance with the Regulations for the Care and Use of Laboratory Animals (311/1997, Ministry of Agriculture, Czech Republic), which is in compliance with the Declaration of Helsinki.

We have demonstrated that ellipticine also forms covalent adducts in DNA after being enzymatically activated with cytochromes P450 (CYP) such as CYP3A4 (Figure 1 and 2A) or peroxidases such as LPO (Figure 2B), MPO (Figure 2C), COX-1 (Figure 2D) and COX-2 (Figure 2E) (Stiborová *et al*., 2001; 2003a; b; 2004; 2006; 2007a; b; Poljaková *et al*., 2006; Moserová *et al*., 2008), suggesting a third possible mechanism of action. Two major DNA adducts generated from 13-hydroxyellipticine (Figure 2F) and 12-hydroxyellipticine (Figure 2G) or *N* ^2^-oxide of ellipticine (Figure 2H), which rearranges to 12-hydroxyellipticine by a Polonowski rearrangement (Stiborová *et al*., 2004), during the ellipticine CYP- and peroxidase-mediated metabolism are formed *in vitro* and *in vivo* in mice (Figure 2I) and rats (Figure 2J) treated with this anticancer drug (Stiborová *et al*., 2001; 2003a; b; 2004; 2006; 2007a; b; 2008; Frei *et al*., 2002; Poljaková *et al*., 2006). The same DNA adducts were also detected in cancer cells in culture, such as human breast adenocarcinoma MCF-7 cells (Figure 2L) (Bořek-Dohalská *et al*., 2004), leukemia HL-60 (Figure 2M) and CCRF-CEM cells (Figure 2N) (Poljaková *et al*., 2007), neuroblastoma cells (Figure 2O) (Poljaková *et al*., 2009) and glioblastoma cells (Figure 2P) (Martínková *et al*., 2009) *in vitro*, and in rat breast adenocarcinoma *in vivo* (Figure 2K) (Stiborová *et al*., 2011). On the basis of these data, ellipticine might be considered a drug, whose pharmacological efficiency and/or genotoxic side effects are dependent on its activation by CYPs and peroxidases in target tissues.

In order to evaluate contributions of DNA adduct formation by ellipticine to its toxicity to cancer cells, a comparison of cytotoxicity and of DNA adduct formation by 0.1–10 µM ellipticine in different cancer cell lines was performed. The ^32^P-postlabeling method was used to determine DNA adduct formation by ellipticine and cytotoxicity of ellipticine was determined with the 3-(4,5-dimethylthiazol-2-yl)-2,5-diphenyl tetrazoliumbromide (MTT) assay (Cinatl *et al*., 1997). Human cancer cell lines sensitive to ellipticine such as human breast adenocarcinoma, leukemia, neuroblastoma and glioblastoma cancer cells were utilized for this study.

## Material and methods

### Chemicals

Ellipticine was obtained from Sigma (St. Louis, MO, USA). All other chemicals used in the experiments were of analytical purity or better.

### Cell cultures

The MCF-7 cell line was from the collection of cell lines of the German Cancer Research Center Heidelberg, Germany). MCF-7 cells were cultivated in Dulbecco's modified Eagle's medium (DMEM, Biochrom AG, Berlin, Germany), high-glucose type (DMEM with 4.5 g D-glucose/l), supplemented with 4 mM L-glutamine, 25 mM HEPES Sigma, St. Louis, MO, U.S.A.), 5% fetal calf serum (Biochrom AG, Berlin, Germany) at 37 °C, 5% CO_2_ and 95% atmospheric humidity.

The U87MG cell line was purchased from ATCC (Manassas, VA, USA). Cells were cultured in Eagle's MEM supplemented with 10% heat-inactivated fetal bovine serum (FBS), 0.6 mg/mL glutamine, 200 international unit (IU)/ml penicillin, 200 IU/ml streptomycin, and 0.1 mg/ml gentamicin (PAA Laboratories, Pasching, Austria) in humidified 5% CO_2_ at 37 °C.

The CCRF-CEM, a T lymphoblastoid cell line, was kindly provided by J.J. McGuire, Roswell Park Cancer Institute, (Buffalo, NY, USA) and HL-60 cells (a promyelotic line) were from the collection of cell lines of the Department of Pediatric Hematology and Oncology, 2^nd^ Medical School, Charles University and University Hospital Motol (Prague, Czech Republic). HL-60 cells were cultivated in Iscove's modified Dulbecco's medium (IMDM, Biochrom AG, Berlin, Germany), high-glucose type, supplemented with 4 mM L-glutamine, 10% fetal calf serum (PAA Laboratories, Pasching, Austria), 100 units (U) per ml of penicillin and 100 µg/ ml streptomycin (PAA,Vienna, Austria) and 0.3% (w/v) NaHCO_3_ at 37 °C, 5% CO_2_ and 95% atmospheric humidity. CCRF-CEM cells were cultivated in RPMI 1640 medium (Biochrom AG,Berlin, Germany) supplemented with 2 mM L-glutamine, 10% fetal calf serum (PAA Laboratories, Pasching, Austria) and 0.3% (w/v) NaHCO3 at 37 °C, 5% CO_2_ and 95% atmospheric humidity.

The UKF-NB-3 and UKF-NB-4 neuroblastoma cell lines, established from bone marrow metastases of high risk neuroblastoma, were a gift of Prof. J. Cinatl, Jr. (J. W. Goethe University, Frankfurt, Germany). The cell line UKF-NB-4 was established from chemoresistant recurrency. IMR-32, high risk neuroblastoma derived cell line, was of commercial source (LGC Promochem, Wesel, Germany). All three cell lines used were derived from high risk neuroblastoma with MYCN amplification, del1p and aneuploidy. Cells were grown at 37 °C and 5% CO_2_ in Iscove's modified Dulbecco's medium (IMDM) (KlinLab Ltd, Prague, Czech Republic), supplemented with 10% fetal calf serum, 2 mM L-glutamine, 100 units/ml of penicilline and 100 µg/ml streptomycine (PAA Laboratories, Pasching, Austria).

### MTT assay

The cytotoxicity of ellipticine was determined by MTT test. For a dose-response curve, solution of ellipticine in dimethyl sulfoxide (DMSO) (1 mM) was dissolved in culture medium to final concentrations of 0–10 µM. Cells in exponential growth were seeded at 1×10^4^ per well in a 96-well microplate. After incubation (48 hours) at 37 °C in 5% CO_2_ saturated atmosphere the MTT solution (2 mg/ml PBS) was added, the microplates were incubated for 4 hours and cells lysed in 50% *N*,*N*-dimethylformamide containing 20% of sodium dodecyl sulfate (SDS), pH 4.5. The absorbance at 570 nm was measured for each well by multiwell ELISA reader Versamax (Molecular devices, CA, USA). The mean absorbance of medium controls was subtracted as a background. The viability of control cells was taken as 100% and the values of treated cells were calculated as a percentage of control. The IC_50_ values were calculated from at least 3 independent experiments using linear regression of the dose-log response curves by SOFTmaxPro.

### Treatment of cancer cell lines with ellipticine for DNA adduct analyses

Cell lines were seeded 24 hr prior to treatment at a density of 1×10^5^ cells/ml in two 75 cm^3^ culture flasks in a total volume of 20 ml of IMDM. Ellipticine was dissolved in 20 µl of DMSO, the final concentration was 0, 0.1, 1, 5 or 10 µM. After 48 h the cells were harvested after trypsinizing by centrifugation at 2 000×g for 3 min and two washing steps with 5 ml of PBS yielded a cell pellet, which was stored at −20 °C until DNA isolation. DNA was isolated and labeled as described in the next section.

### DNA isolation and ^32^P-postlabeling of DNA adducts

DNA from cells was isolated by the phenol-chloroform extraction as described (Frei *et al*., 2002; Bořek-Dohalská *et al*., 2004; Poljaková *et al*., 2007; 2009). ^32^P-postlabeling analyses were performed using nuclease P1 enrichment as described previously (Stiborová *et al.*, 2001) From experiments performed earlier, calf thymus DNA incubated with ellipticine activated with CYP3A4 (Stiborová *et al*., 2004), LPO, MPO, COX-1 and -2 (Stiborová *et al*., 2007a), with 13-hydroxyellipticine, 12-hydroxyellipticine and *N* ^2^-oxide of ellipticine (Stiborová *et al*., 2004; 2007a) and liver DNA of rats (Stiborová *et al*., 2007b) or mice (Stiborová *et al*., 2008) treated with ellipticine were labeled with ^32^P to compare adduct spot patterns.

### HPLC analysis of ^32^P-labeled DNA adducts

HPLC analysis was performed essentially as described previously (Stiborová *et al*., 2003a; b; 2004). Individual spots detected by ^32^P-postlabeling were excised from the thin layer and extracted (Stiborová *et al*., 2003a; b; 2004). Cut-outs were extracted with two 800 µl portions of 6 M ammonium hydroxide/isopropanol (1:1) for 40 min. The eluent was evaporated in a Speed-Vac centrifuge. The dried extracts were dissolved in 100 µl of methanol/phosphate buffer (pH 3.5) 1:1 (v/v). Aliquots (50 µl) were analyzed on a phenyl-modified reversed-phase column (250 mm×4.6 mm, 5 µm Zorbax Phenyl; Säulentechnik Knauer, Berlin, Germany) with a linear gradient of methanol (from 40 to 80% in 45 min) in aqueous 0.5 M sodium phosphate and 0.5 M phosphoric acid (pH 3.5) at a flow rate of 0.9 ml/min. Radioactivity eluting from the column was measured by monitoring Cerenkov radiation with a Berthold LB 506 C-I flow-through radioactivity monitor (500 µl cell, dwell time 6 s).

## Results

### Cytotoxicity of ellipticine to human breast adenocarcinoma MCF-7, leukemia HL-60 and CCRF-CEM cells, neuroblastoma IMR-32, UKF-NB-3 and UKF-NB-4 cells, and glioblastoma U87MG cells

To determine the cytotoxicity of ellipticine to human breast adenocarcinoma MCF-7, leukemia HL-60 and CCRF-CEM cells, neuroblastoma IMR-32, UKF-NB-3 and UKF-NB-4 cells and glioblastoma UKF87MG cells, they were treated with increasing concentrations of ellipticine and viable cells detected with MTT assay. As shown in Table 1, all cell lines were sensitive to ellipticine. Ellipticine inhibited the growth of all cell lines tested in this study in a dose-dependent manner. The IC_50_ values for ellipticine calculated from the dose-log response curves are shown in Table 1. Neuroblastoma IMR-32 cells, followed by neuroblastoma UKF-NB-4 and UKF-NB-3 and leukemia HL-60 cells, were the cells most sensitive to ellipticine, with IC_50_ values lower than 1 µM (see IC_50_ values shown in Table 1). When sensitivity of additional cells to ellipticine was compared, cytotoxicity of this agent to human breast adenocarcinoma MCF-7 cells and a glioblastoma U87MG cell line was found to be comparable (the IC_50_ values were around 1 µM), while leukemia CCRF-CEM cells were less sensitive. The IC_50_ value for ellipticine was almost 4-times higher in these leukemia cells than in MCF-7 and U87MG cells (Table 1).

**Table 1:** Cytotoxicity of ellipticine to human cancer cell lines.

Cells	IC_50_ (µM)
Breast adenocarcinoma MCF-7	1.25±0.13
Leukemia HL-60	0.67±0.06
Leukemia CCRF-CEM	4.70±0.48
Neuroblastoma IMR-32	0.27±0.02
Neuroblastoma UKF-NB-3	0.44±0.03
Neuroblastoma UKF-NB-4	0.49±0.04
Glioblastoma U87MG	1.48±0.62

IC_50_ values were calculated from the linear regression of the dose-log response curves. Values are mean±S.D. of at least 3 experiments.

### Determination of DNA adduct formation by ellipticine in human breast adenocarcinoma MCF-7, leukemia HL-60 and CCRF-CEM cells, neuroblastoma IMR-32, UKF-NB-3 and UKF-NB-4 cells, and glioblastoma U87MG cells

The cell lines shown to be sensitive to ellipticine (Table 1) were treated with increasing concentrations of ellipticine (0.1–10 µM) for 48 h and DNA was isolated from these cells. Using the nuclease P1 version of ^32^P-postlabeling assay, which was found to be suitable to detect and quantify DNA adducts formed by ellipticine (Stiborová *et al*., 2001; 2003a; b; 2004; 2007a; b; 2008; 2011), ellipticine-derived adducts were detected in the DNA of these cells (Figure 2L-P, Table 2). Two major ellipticine-DNA adducts (spots 1 and 2 in Figure 2) were formed in all cells (Table 2). No adducts were detected in DNA of control cells treated with solvent only.

**Table 2:** DNA adduct formation by ellipticine in human cancer cell lines.

	Levels of DNA adducts (RALx10^−7^)^a^				
Cells	Adduct 1	Adduct 2	Adduct 6	Adduct 7	Total
**MCF-7**					

+ 1.0 µM ellipticine	0.12±0.01	0.20±0.02	n.d.	n.d.	0.32±0.03
+ 5 µM ellipticine	1.90±0.20	2.63±0.30	0.17±0.02	n.d.	4.70±0.50
+ 10 µM ellipticine	3.72±0.40	4.77±0.50	0.81±0.07	n.d.	9.30±0.92

**HL-60**					

+ 0.1 µM ellipticine	0.41±0.04	0.24±0.01	n.d.	n.d.	0.65±0.07
+ 1.0 µM ellipticine	4.30±0.42	3.20±0.34	n.d.	n.d.	7.50±0.73
+ 5 µM ellipticine	46.32±4.30	21.18±2.30	n.d.	n.d.	67.50±6.23

**CCRF-CEM**					

+ 0.1 µM ellipticine	0.02±0.01	0.01±0.01	n.d.	n.d.	0.03±0.01
+ 1.0 µM ellipticine	1.12±0.01	1.08±0.02	n.d.	n.d.	2.20±0.22
+ 5 µM ellipticine	3.60±0.30	3.20±0.40	n.d.	n.d.	6.80±0.65
+ 10 µM ellipticine	9.40±0.95	8.40±0.79	n.d.	n.d.	17.80±1.62

**IMR-32**					

+ 0.1 µM ellipticine	0.10±0.01	0.13±0.01	n.d.	n.d.	0.23±0.02
+ 1.0 µM ellipticine	0.26±0.02	0.31±0.03	n.d.	n.d.	0.57±0.05
+ 10 µM ellipticine	13.15±1.30	13.13±1.30	n.d.	n.d.	26.28±2.60

**UKF-NB-3**					

+ 1.0 µM ellipticine	0.12±0.01	0.23±0.02	n.d.	n.d.	0.35± 0.04
+ 10 µM ellipticine	3.26±0.32	2.64±0.40	n.d.	n.d.	5.90±0.68

**UKF-NB-4**					

+ 0.1 µM ellipticine	0.04±0.01	0.01±0.01	n.d.	n.d.	0.05±0.01
+ 1.0 µM ellipticine	0.20±0.02	0.38±0.04	n.d.	n.d.	0.58±0.06
+ 10 µM ellipticine	5.40±0.56	6.50±0.81	0.27±0.03	0.37±0.05	12.54±1.51

**U87MG**					

+ 1.0 µM ellipticine	0.11±0.01	0.19±0.02	n.d.	n.d.	0.30± 0.04
+ 10 µM ellipticine	1.98±0.15	3.42±0.33	n.d.	n.d.	5.40±0.53

Cancer cells were exposed to ellipticine for 48 h. DNA adducts were analyzed by the nuclease P1 version of the ^32^P-postlabeling assay. ^*a*^RAL, relative adduct labeling; averages and S.D. of three experiments. n.d.-not detected (the detection limit of RAL was 1/10^10^ nucleotides).

Chromatographic properties of the two major adduct spots on PEI-cellulose TLC plates (spots 1 and 2) were similar to those of ellipticine-derived DNA adducts found previously after *in vitro* incubation of calf thymus DNA with ellipticine and isolated CYPs (Stiborová *et al*., 2001; 2003b; 2004), or peroxidases (Stiborová *et al*., 2007a) or *in vivo* (Figure 2D), in several organs of rats (Stiborová *et al*., 2003a; 2007b) and mice (Stiborová *et al*., 2008) exposed to this agent. Both these adducts were found to be generated from 13-hydroxy- and 12-hydroxyellipticine (Figure 2E,F), as confirmed by cochromatographic analysis using TLC and HPLC (data not shown). Both adducts were identified as deoxyguanosine adducts in DNA (Stiborová *et al*., 2003b; Moserová *et al*., 2008)). Besides these adducts, additional two minor adducts (spots 6 and 7 in Figure 5C,D) were detected in DNA of MCF-7 (spot 6) and UKF-NB-4 (spots 6 and 7) cells treated with 10 µM ellipticine (Table 2, Figure 2). Both these minor adducts are known to be generated *in vitro* mainly by peroxidase-catalyzed oxidation (Poljaková *et al*., 2006; Stiborová *et al*., 2007a). The low levels of these adducts prevented HPLC co-chromatographic analysis or their further characterization.

Ellipticine-DNA adduct levels were dose dependent in all cells with an overproportional increase between 1 µM and 10 µM (or 5 µM) ellipticine in MCF-7, HL-60, IMR-32, UKF-NB-3, UKF-NB-4 and U87MG cells, but not in CCRF-CEM cells (Table 2). The highest levels were formed in leukemia HL-60 cells (Table 2).

## Discussion

The results of this study show a comparison of ellipticine cytotoxicity to several human cancer cell lines (breast adenocarcinoma MCF-7, leukemia HL-60 and CCRF-CEM cells, neuroblastoma IMR-32, UKF-NB-3, UKF-NB-4 lines and glioblastoma U87MG cells). In addition, the mechanism of ellipticine cytotoxicity to these cells was evaluated. The mode of antitumor, cytotoxic and mutagenic action of ellipticine is considered to be based mainly on DNA damage, such as intercalation into DNA, inhibition of topoisomerase II, and formation of covalent DNA adducts mediated by CYPs and peroxidases (Auclair, 1987, Stiborová *et al*., 2001; 2006; 2011; Garbett & Graves, 2004). Intercalation of ellipticine into DNA and inhibition of topoisomerase II occur in all cell types irrespective of their metabolic capacity, because of the general chemical properties of this drug and its affinity to DNA and topoisomerase II protein (Auclair, 1987). However, the formation of ellipticine-DNA adducts, which is dependent on ellipticine activation by CYPs and peroxidases, has not yet been proven as a general mechanism. we found this ellipticine action unambiguously *in vitro*, using several CYP and peroxidase enzymes for ellipticine activation (Stiborová *et al*., 2001; 2003a; b; 2004; 2007a) and *in vivo* in rats and mice (Stiborová *et al*., 2003a; 2007b; 2008). In our former studies (Bořek-Dohalská *et al*., 2004; Poljaková *et al*., 2007; 2009; Martínková *et al*., 2009) and in the present work, ellipticine-DNA adducts were detected also in several human cancer cell lines. Here we evaluated a contribution of this mechanism to ellipticine toxicity to these cancer cells.

Toxic effects of ellipticine to leukemia HL-60 and CCRF-CEM cells (expressed as IC_50_ values) correspond to levels of ellipticine-DNA adducts formed in these cells. This finding indicates that covalent modification of DNA by ellipticine plays an important role in cytotoxicity of ellipticine to these leukemia cells. Even though the IC_50_ values for ellipticine in MCF-7 and U87MG cells essentially correspond to levels of DNA adducts formed by ellipticine, we cannot account these data as valuable because these cell lines are cells of different cancer types. In the case of two of neuroblastoma cell lines tested, IMR-32 and UKF-NB-4, toxic effects of ellipticine to these cells also correspond to levels of ellipticine-DNA adducts formed in these cells. The cytotoxic activity of ellipticine to IMR-32 and UKF-NB-4 neuroblastoma cell lines was also previously found to be a consequence of the formation of ellipticine-DNA adducts (Poljaková *et al*., 2009). In addition, the role of ellipticine-DNA adduct formation in cytotoxicity of this drug to neuroblastoma cells was further supported by the finding that a decrease in the levels of these adducts in IMR-32 and UKF-NB-4 cells under hypoxic conditions correlated with a decrease in toxicity of ellipticine under these conditions (Poljaková *et al*., 2009). This is, however, not the case of the UKF-NB-3 cell line; lower levels of DNA adducts were found in these cells than in UKF-NB-4 cells, although both neuroblastoma cells exhibited similar sensitivity to ellipticine. All these findings suggest that DNA adduct formation by ellipticine might be the predominant mechanism responsible for ellipticine cytotoxicity to most of cancer cell lines tested in this work, except UKF-NB-3 neuroblastoma cells. Thus the DNA adduct formation by ellipticine is probably not the major mechanism responsible for ellipticine cytotoxicity to UKF-NB-3 neuroblastoma cells. Other mechanisms such as intercalation into DNA (Auclair, 1987; Singh *et al*., 1994) and inhibition of DNA topoisomerase II activity (Auclair, 1987; Monnot *et al*., 1991; Fossé *et al*., 1992; Froelich-Ammon *et al*., 1995) that were found to be additional DNA-mediated mechanisms of ellipticine antitumor, mutagenic and cytotoxic activities [for a summary see (Stiborová et al., 2001; 2006; 2011)] seem to contribute to ellipticine cytotoxicity to these neuroblastoma cells. In order to resolve the real contribution of covalent DNA adduct formation by ellipticine in these cells, additional DNA damage such as intercalation of ellipticine into DNA in these and other cancer cells should be assessed. Recently, we used square wave voltametry to evaluate intercalation of ellipticine into DNA *in vitro* and covalent modification of DNA by ellipticine in a UKF-NB-3 neuroblastoma cell line and found participation of both these mechanisms in DNA damage caused by this drug in this neuroblastoma cell line (Huska *et al*., 2010a; Huska *et al*., 2010b). Nevertheless, further detailed studies have to be performed which would examine differences in electrochemical signals of DNA with intercalated ellipticine and DNA covalently modified by this antitumor agent.

In our former studies, toxic effects of ellipticine to several cancer cells were found to be dependent on expression of CYP1A1, 1B1, 3A4 and peroxidases LPO, COX and MPO in these cells (Bořek-Dohalská *et al*., 2004; Poljaková *et al*., 2007; 2009; Martínková *et al*., 2009). Moreover, expression of CYP1A1, 1B1 and 3A4 enzymes was found to be induced by treating glioblastoma U87MG, adenocarcinoma MCF-7 and neuroblastoma UKF-NB-4 cells with ellipticine (Martínková *et al*., 2009; Stiborová *et al*., unpublished results). Likewise, CYP1A1 was found to be induced by ellipticine in rats *in vivo* (Aimová *et al*., 2007). Hence, the sensitivity of individual cancer cells to ellipticine caused by covalent modification of DNA might be dependent on the capability of ellipticine to induce the CYP enzymes oxidizing ellipticine to 13-hydroxy- and 12-hydroxyellipticine, the metabolites generating DNA adducts. This suggestion, however, needs to be confirmed by further investigations including *in vivo* studies. This feature is one of the aims of investigations in our laboratory.
